# A Standardized Framework for Better Understanding of Phenotypic Differences within Bacterial Phyla Based on Protein Domain

**DOI:** 10.1128/jb.00141-22

**Published:** 2022-06-02

**Authors:** Boqian Wang, Jianglin Zhou, Yuan Jin, Mingda Hu, Yunxiang Zhao, Xin Wang, Long Liang, Junjie Yue, Hongguang Ren

**Affiliations:** a Beijing Institute of Biotechnology, State Key Laboratory of Pathogen and Biosecurity, Beijing, China; b Beijing Institute of Microbiology and Epidemiology, State Key Laboratory of Pathogen and Biosecurity, Beijing, China; Queen Mary University of London

**Keywords:** protein domain, bacterial kingdom, framework of classification, phenotypic or genotypic differences

## Abstract

We propose a standardized framework to classify target species based on their protein domains, which can be utilized in different contexts, like eukaryotes and prokaryotes. In this study, by applying the framework to the bacterial kingdom as an implementation example and comparing the results with the current taxonomy standards at the phylum level, we came to the conclusion that the sequence of domains rather than the content of domains in a protein and the presence of one domain rather than the number of occurrences of one domain play more important roles in deciding bacterial phenotypes as well as matching the current taxonomy. In addition, the comparison also helps us to better focus on the species that conflict with the current phylum category, as well as to further investigate their phenotypic or genotypic differences.

**IMPORTANCE** A 3-step framework was designed which can be applied to clustering species based on their protein domains, and different candidate models are proposed in each step for better adaptation of various scenarios. We show its implementation for the bacterial kingdom as an example, which helps us to find the most appropriate model combination that will best reflect the relationship between domains and phenotypes in this context. In addition, identifying species that are distant in the results but should be closely related phylogenetically can help us to focus on the mismatch for better understanding of their key phenotypic or genotypic differences.

## INTRODUCTION

With the development of biotechnology, genetic information offers us the possibility to look into the essence of classification as well as phenotypic or genotypic differences among species.

The utilization of the genome sequence is an intuitive method which directly focuses on the most original and basic data (1, 2). It can be applied to scenarios requiring fine-grained analysis, for example, the detection of the gene mutation or recombination events in coronavirus (3, 4). The protein sequence is similar to the genome sequence, with the exception of masking some gene-level differences during codon translation (5, 6).

Comparatively, the protein domain, as the basic functional unit of protein, is an ideal bridge that can connect the genetic sequence with biological function (7–9). A protein usually contains one or many domains, each of which consists of 50 to 350 amino acids. The domain has a direct relationship with protein function and thus with phenotypes, which can be utilized as a standard of classification.

Nowadays, much research is conducted based on protein domains (7–10). However, several important problems remain to be solved. First, a systematic and automatic method to deal with domain information for classification and comparison is still lacking. Second, how to utilize the domain data to better reflect phenotypic or genotypic similarities and differences in different species is still not clear.

In this study, to solve these problems, we developed a standardized framework to systematically support classification of species based on the protein domain, which will be fully automatized in future work. The framework consists of three steps to separately collect the number of domains in each species, calculate the distance of each pair of species, and realize classification based on the minimum-cost spanning tree (MST). Different statistical models are involved in the first and second steps as candidates to fulfill requirements of different research targets.

As an implementation example, we applied the framework to 2,568 selected species from the bacterial kingdom and compared the results with bacterial taxonomy at the phylum level. We found the best combination of models in the framework, and we discuss the reflected biological significance. In addition, the results validate our proposed framework. Finally, phenotypic or genotypic differences within established phyla were investigated and are discussed.

## RESULTS

**FIG 1:**
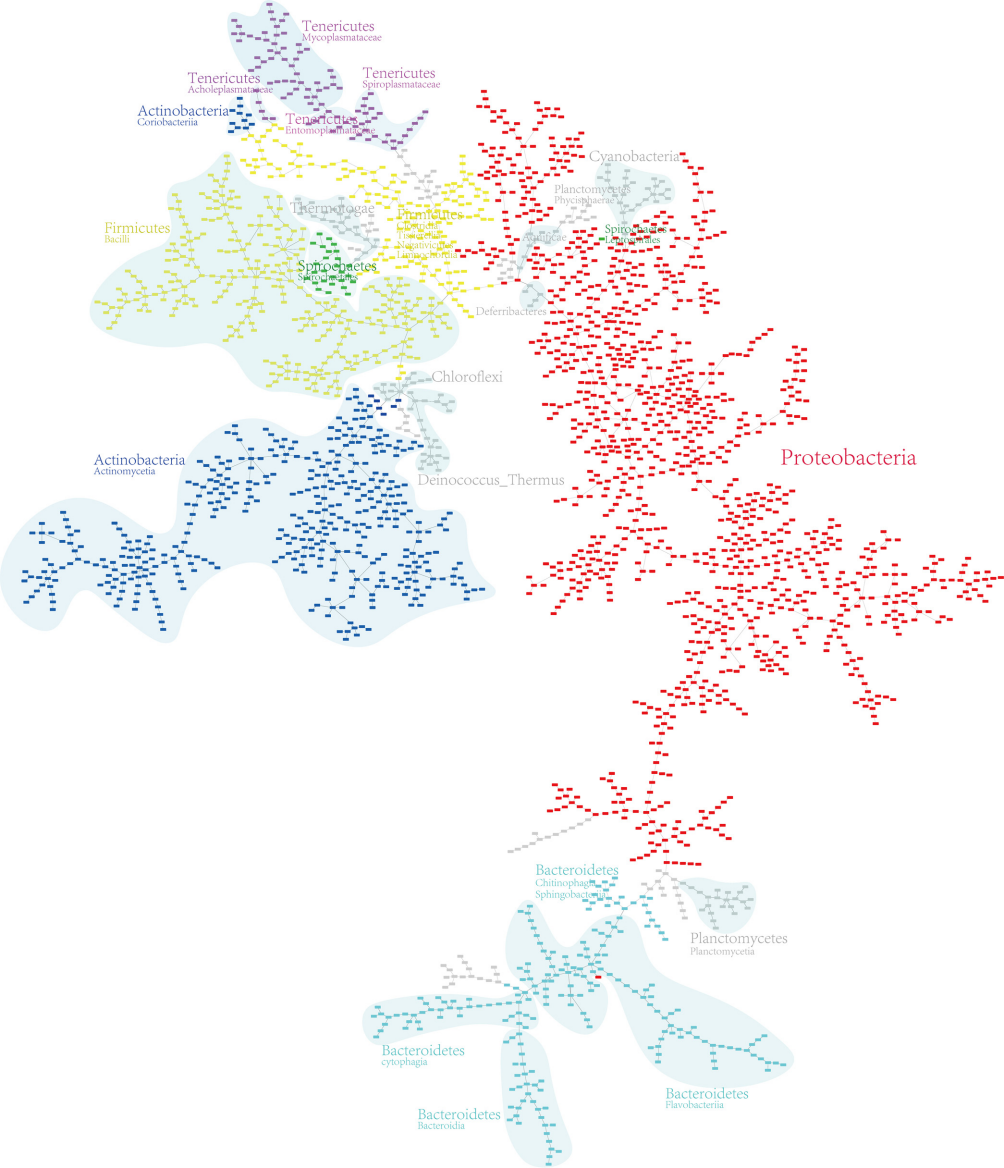
MST result obtained with the “content” model and the Jaccard distance model. The MST result is manually preprocessed by grouping species at the phylum and class levels, and the phyla including more than 10 species are distinguished with different colors.

Twelve MST results (https://github.com/wr-sky/Domain-Bac-Tax/tree/main/7-data/json_edgelist) are generated by 12 different combinations of methods in the framework and visualized by Cytoscape (11). Detailed results can be found in Fig. S1 to S12 in the supplemental material. Taking the “content” model with Jaccard distance as an example, we display the MST result in Fig. 1.

For ease of analysis, we connected GCF (A RefSeq genome assembly derived by NCBI. Each genome assembly in NCBI is assigned with an unique GCF number.) and taxonomy information in a file (https://github.com/wr-sky/Domain-Bac-Tax/tree/main/7-data/gcf_taxonomy), which can be read by Cytoscape to color each node automatically. The colored area and taxonomy text in the figure were manually marked to show the classification more clearly. Obviously, the results match the National Center for Biotechnology Information (NCBI) taxonomy very well.

The clustering results corresponding to the 12 MST results can be found in Tables S1 and S2. Table S1 shows the results by Jaccard and Poisson distance models in terms of phylum, listing the number of species in each group that was isolated from its main part (the group with the maximal number of species). If all species of a phylum are included in a group, the corresponding grid will remain blank.

Since the loss-corrected distance model results in too many isolated small groups for some phyla, Table S2 records only the number of groups that each phylum has been isolated into by the loss-corrected distance model.

To compare the results more comprehensively, we propose three standards of measurement: the percentage of isolated species (arithmetic percentage), the weighted percentage of isolated species (weighted percentage), and the number of phyla being divided into more than one group (phylum number). In equations 1 and 2, *S_i_* stands for the number of species that are isolated from their main group in each phylum and *T_i_* stands for the species number of each phylum. The parameter *i* ranges from 1 to 31, representing 30 bacterial phyla and 1 archaeal domain.

(1) $$\text{arithmetic percentage}=\overset{31}{\underset{i=1}{\sum }}S_{i}÷\overset{31}{\underset{i=1}{\sum }}T_{i}$$

(2) $$\text{weighted percentage}=\overset{31}{\underset{i=1}{\sum }}(\frac{S_{i}}{T_{i}})÷31$$

**FIG 2:**
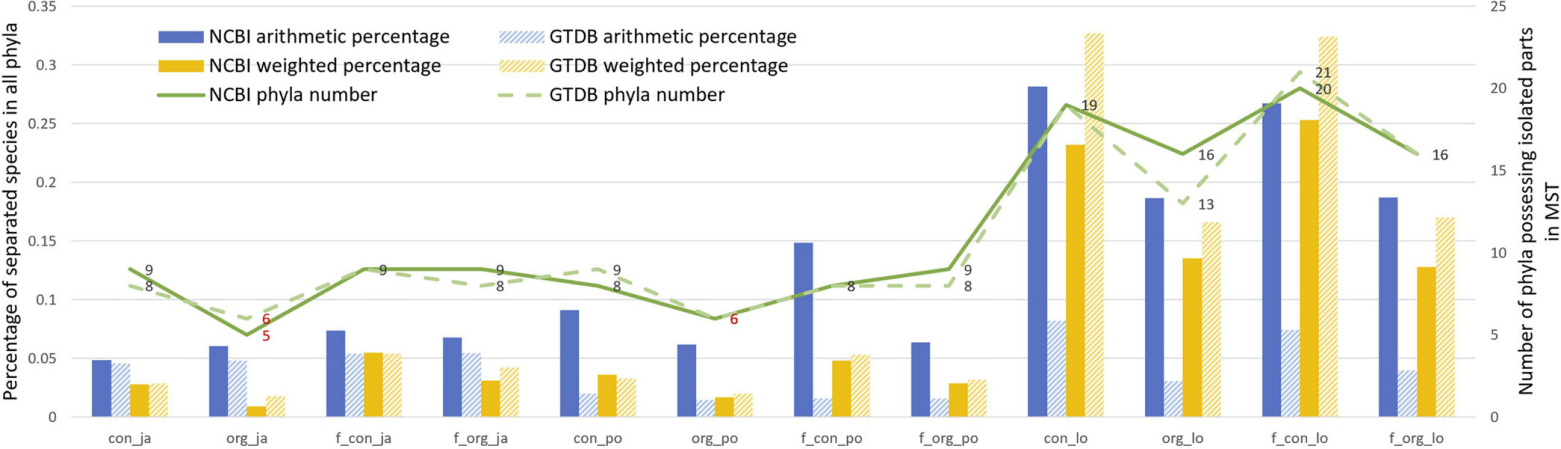
Comparison of the twelve methods with NCBI and GTDB taxonomies by three different standards. Con, org, f_con, and f_org represent the “content,” “organization,” “f_content,” and “f_organization” models, respectively. ja, po, and lo stand for Jaccard, Poisson, and loss-corrected distance models, respectively.

We show the comparison results in Fig. 2 and additionally involve the GTDB (12, 13) taxonomic classification of prokaryotes for comparison (https://github.com/wr-sky/Domain-Bac-Tax/tree/main/7-data/gcf_taxonomy). We have marked the best and the second-best solutions for each standard with red font. The conclusion can be summarized as follows. (i) The “organization” model is better than the other three models (“content,” “f_content,” and “f_organization”) in most cases when three different distance methods are used. It illustrates that the sequence of domains rather than the content of domains in a protein and the presence of one domain rather than the number of instances of one domain play more important roles in deciding bacterial phenotypes as well as matching the current taxonomy. (ii) The Jaccard and Poisson methods are better that the loss-corrected method, which means that the Jaccard and Poisson methods reflect the bacterial relationship more precisely in terms of the domain. (iii) The NCBI results are better than the GTDB results when weighted percentage is used as the standard, while the GTDB results are better than the NCBI results when arithmetic percentage is used as the standard. The difference is caused by the phyla with a smaller number of species, which will be more influential in the standard of arithmetic percentage and thus more suitable for GTDB taxonomy.

## DISCUSSION

**FIG 3:**
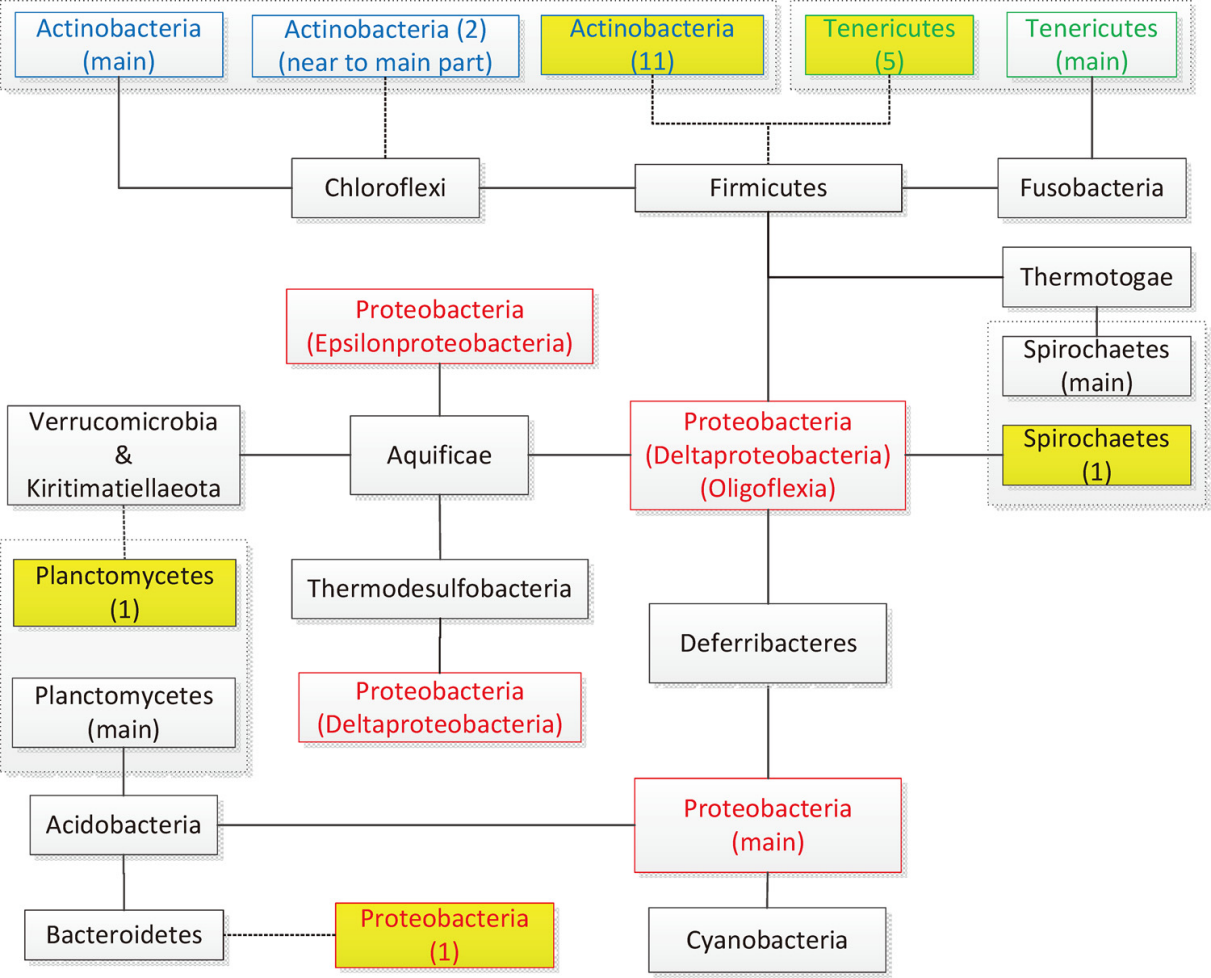
Abstract representation of an MST generated by the org_ja method. Six groups of species separated from their main phylum are connected to their neighboring phyla by dashed lines. Five of them are far from their main phylum (yellow background), and the numbers of species are given.

To show the classification result more clearly, we simplified the MST generated by the org_ja model combination in Fig. 3. It is obvious that five groups of species are widely separated from their main phylum (yellow background in Fig. 3): *Actinobacteria* (*Coribobacteriia*), *Tenericutes* (*Acholeplasmatales*), *Spirochaetes* (*Leptospirales*), *Planctomycetes* (*Phycisphaerae*), and *Proteobacteria* (Glaciecola amylolytica). This indicates a relatively high degree of protein domain differences and probably phenotypic differences between species in these five groups (19 species) and their corresponding main phylum.

The mismatch of the classification result with the taxonomy prompted us to look into details of the specific species, which is a way of utilizing the proposed framework. The taxonomy information of these 19 species is listed in Table 1. Their genotypic and phenotypic differences are discussed below. We found that consistency with related works can also validate the framework.

**TABLE 1:** Classification of the 19 species utilized here^*a*^

GCF	Phylum	Class	Order	Family	Genus	Species
900169485	*Actinobacteria*	***Coriobacteriia***	*Eggerthellales*	*Eggerthellaceae*	*Phoenicibacter*	*Phoenicibacter congonensis*
000023845	*Actinobacteria*	***Coriobacteriia***	*Eggerthellales*	*Eggerthellaceae*	*Cryptobacterium*	Cryptobacterium curtum
900637575	*Actinobacteria*	***Coriobacteriia***	*Eggerthellales*	*Eggerthellaceae*	*Slackia*	Slackia heliotrinireducens
001643775	*Actinobacteria*	***Coriobacteriia***	*Eggerthellales*	*Eggerthellaceae*	*Denitrobacterium*	Denitrobacterium detoxificans
000024265	*Actinobacteria*	***Coriobacteriia***	*Eggerthellales*	*Eggerthellaceae*	*Eggerthella*	Eggerthella lenta
000478885	*Actinobacteria*	***Coriobacteriia***	*Eggerthellales*	*Eggerthellaceae*	*Adlercreutzia*	Adlercreutzia equolifaciens
000195315	*Actinobacteria*	***Coriobacteriia***	*Coriobacteriales*	*Coriobacteriaceae*	*Coriobacterium*	Coriobacterium glomerans
900119915	*Actinobacteria*	***Coriobacteriia***	*Coriobacteriales*	*Atopobiaceae*	*Olsenella*	*Olsenella timonensis*
000143845	*Actinobacteria*	***Coriobacteriia***	*Coriobacteriales*	*Atopobiaceae*	*Olsenella*	Olsenella uli
000024225	*Actinobacteria*	***Coriobacteriia***	*Coriobacteriales*	*Atopobiaceae*	*Lancefieldella*	*Lancefieldella parvula*
003966955	*Actinobacteria*	***Coriobacteriia***	*Coriobacteriales*	*Atopobiaceae*	*Parolsenella*	*Parolsenella catena*
000967915	*Tenericutes*	*Mollicutes*	***Acholeplasmatales***	*Acholeplasmataceae*	*Acholeplasma*	Acholeplasma brassicae
900660755	*Tenericutes*	*Mollicutes*	***Acholeplasmatales***	*Acholeplasmataceae*	*Acholeplasma*	Acholeplasma hippikon
000018785	*Tenericutes*	*Mollicutes*	***Acholeplasmatales***	*Acholeplasmataceae*	*Acholeplasma*	Acholeplasma laidlawii
000968055	*Tenericutes*	*Mollicutes*	***Acholeplasmatales***	*Acholeplasmataceae*	*Acholeplasma*	Acholeplasma palmae
900660745	*Tenericutes*	*Mollicutes*	***Acholeplasmatales***	*Acholeplasmataceae*	*Acholeplasma*	Acholeplasma axanthum
000266885	*Spirochaetes*	*Spirochaetia*	***Leptospirales***	*Leptospiraceae*	*Turneriella*	Turneriella parva
001999965	*Planctomycetes*	***Phycisphaerae***	*Sedimentisphaerales*	*Sedimentisphaeraceae*	*Limihaloglobus*	*Limihaloglobus sulfuriphilus*
003856375	*Proteobacteria*	*Gammaproteobacteria*	*Alteromonadales*	*Alteromonadaceae*	*Glaciecola*	***Glaciecola amylolytica***

a The 19 species include 11 species in *Actinobacteria*, five species in *Tenericutes*, and one species each in *Planctomycetes*, *Proteobacteria*, and *Spirochaetes*. The 11 *Actinobacteria* species are separated from the main *Actinobacteria* species at the class level, which means if and only if the species in class *Coriobacteriia* are separated from the species in the other classes of the phylum *Actinobacteria*. The separation level of each phylum is indicated by boldface type.

### Actinobacteria.

Four hundred thirty-two examples of *Actinobacteria* are included in the data set, and 11 species in the class *Coriobacteriia* are isolated from the other classes, the neighboring *Firmicutes* (*Erysipelotrichia*). This indicates that species in *Coriobacteriia* may have significant phenotypic differences from the other classes in *Actinobacteria*.

This topic has also been discussed in other works. The identification of a number of conserved signature indels (CSIs) and conserved signature proteins (CSPs) shows that they are commonly and uniquely shared by the most members of all other classes of *Actinobacteria* except *Coriobacteriia*, which branches more deeply. It indicates the possibility of excluding *Coriobacteriia* from *Actinobacteria* (14). This conclusion is also emphasized by another work, which proposed that the species Symbiobacterium thermophilum should be moved from *Actinobacteria* to *Firmicutes* on the basis of CSI and CSP standards and the genome sequence, as well as other lines of evidence (15, 16).

### Tenericutes.

Ninety-four examples of *Tenericutes* are included in the data set, and five species in the order *Acholeplasmatales* are isolated from the other orders, connecting to *Firmicutes* (*Erysipelotrichia*).

From the perspective of phenotype, the species in the order *Acholeplasmatales* do not require sterol for growth, which is quite different from species in the other orders, resulting in a distant relationship between them (17). In addition, from the perspective of taxonomy, *Tenericutes* has belonged to *Firmicutes* (18). Therefore, it is reasonable that *Acholeplasmatales* is near *Firmicutes* in our MST result. Interestingly, in GTDB taxonomy, the order *Acholeplasmatales* has already been moved back to *Firmicutes* in the class *Bacilli*.

### Spirochaetes.

Twenty-eight examples of the order *Spirochaetes* are included in the data set, and one species in the order *Leptospirales* is separated from the *Spirochaetales*, connecting to *Proteobacteria* (*Oligoflexia*).

**FIG 4:**
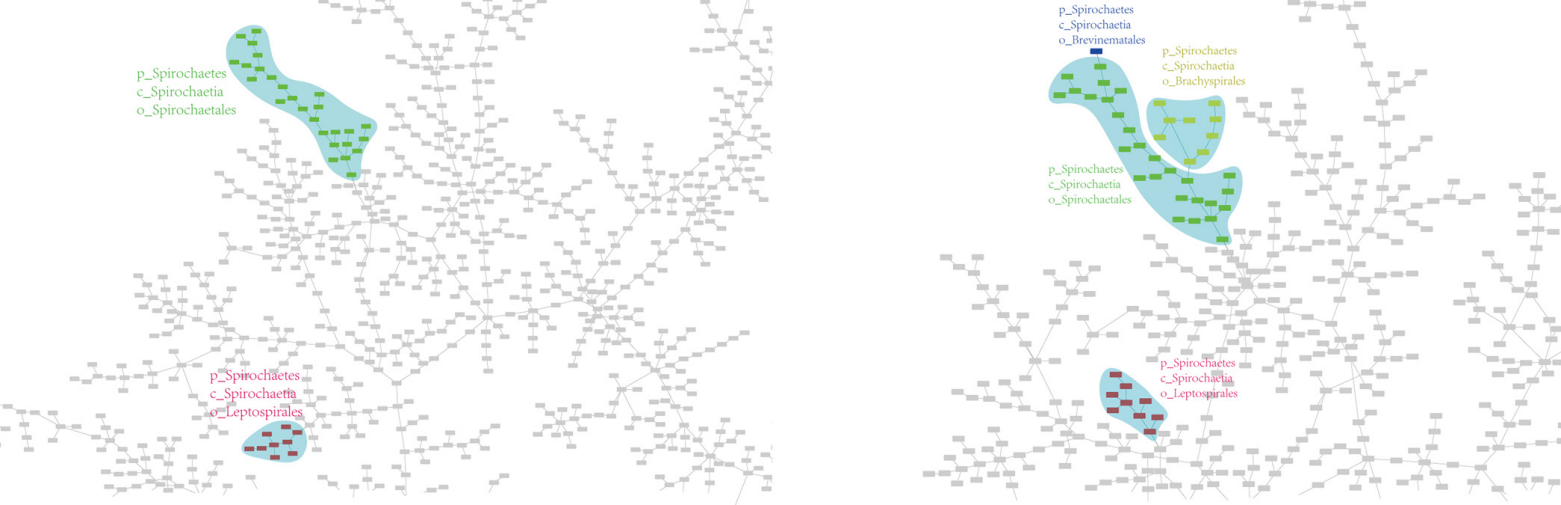
Eight species in the order *Leptospirales* were added to generate the MST (left). Another nine species in *Brachyspirales* and one species in *Brevinematales* were included to explore the relationship of the four orders in *Spirochaetes* (right).

To erase the deviation caused by the single example, we randomly analyzed another 8 species in *Leptospirales*. Their detailed information is listed in Data Set S2. The updated MST is shown in Fig. 4 (left), where it is seen that *Leptospirales* is still separated from *Spirochaetales*.

From the perspective of taxonomy, there are two other orders in *Spirochaetes*: *Brachyspirales* and *Brevinematales*. Therefore, we further analyzed species in these orders to recreate the MST with the org_ja method. The result is shown in Fig. 4 (right), and the detailed information is listed in Data Set S2. It is clearly shown in the figure that *Leptospirales* is separated from the other three orders, which closely connect to each other.

We collected the phenotypes of these orders in Table 2 (19). *Leptospirales* has the phenotypes of hooked-end flagella, not overlapping periplasmic flagella, and an aerobic habit, which are different from the phenotypes of species in the other orders. In GTDB taxonomy, the order *Leptospirales* has been promoted to a new class, *Leptospirae*, in the *Spirochaetes*.

**TABLE 2:** Comparison of four characteristics of five orders^*a*^

Order	Shape	Hooked ends	Periplasmic flagella	Habit
*Spirochaetales*	Helical, coccoid	No	Central, overlapped	Anaerobic, microaerophilic
*Brachyspirales*	Helical	No	Central, overlapped	Anaerobic
*Brevinematales*	Helical	No	Central, overlapped	Microaerophilic
*Leptospirales*	Helical	Yes	Central, not overlapped	Aerobic, microaerophilic

a Four orders in the phylum *Spirochaetes* share the same helical cell shape. Species in the order *Leptospirales* have no hooked ends, which is different from species in the other three orders in the phylum *Spirochaetes*. In addition, the periplasmic flagella of *Spirochaetales*, *Brachyspirales*, and *Brevinematales* overlap and are located in the central region of the cell. Finally, species in *Leptospirales* are aerobic or at least microaerophilic, which is not the case in the other three orders in *Spirochaetes*, where species are usually anaerobic or microaerophilic at most. As for the order *Oligoflexia* in the phylum *Proteobacteria*, it is also quite different from *Leptospirales* with regard to these characteristics.

### Planctomycetes.

Twenty-three examples of *Planctomycetes* are included in the data set, and one species of the class *Phycisphaerae* is separated from the class *Planctomycetia*, neighboring *Kiritimatiellaeota*. In contrast, these two classes are located very near each other according to the “f_content” and “f_organization” models combined with the Jaccard distance model (f_con_ja and f_org_ja).

**FIG 5:**
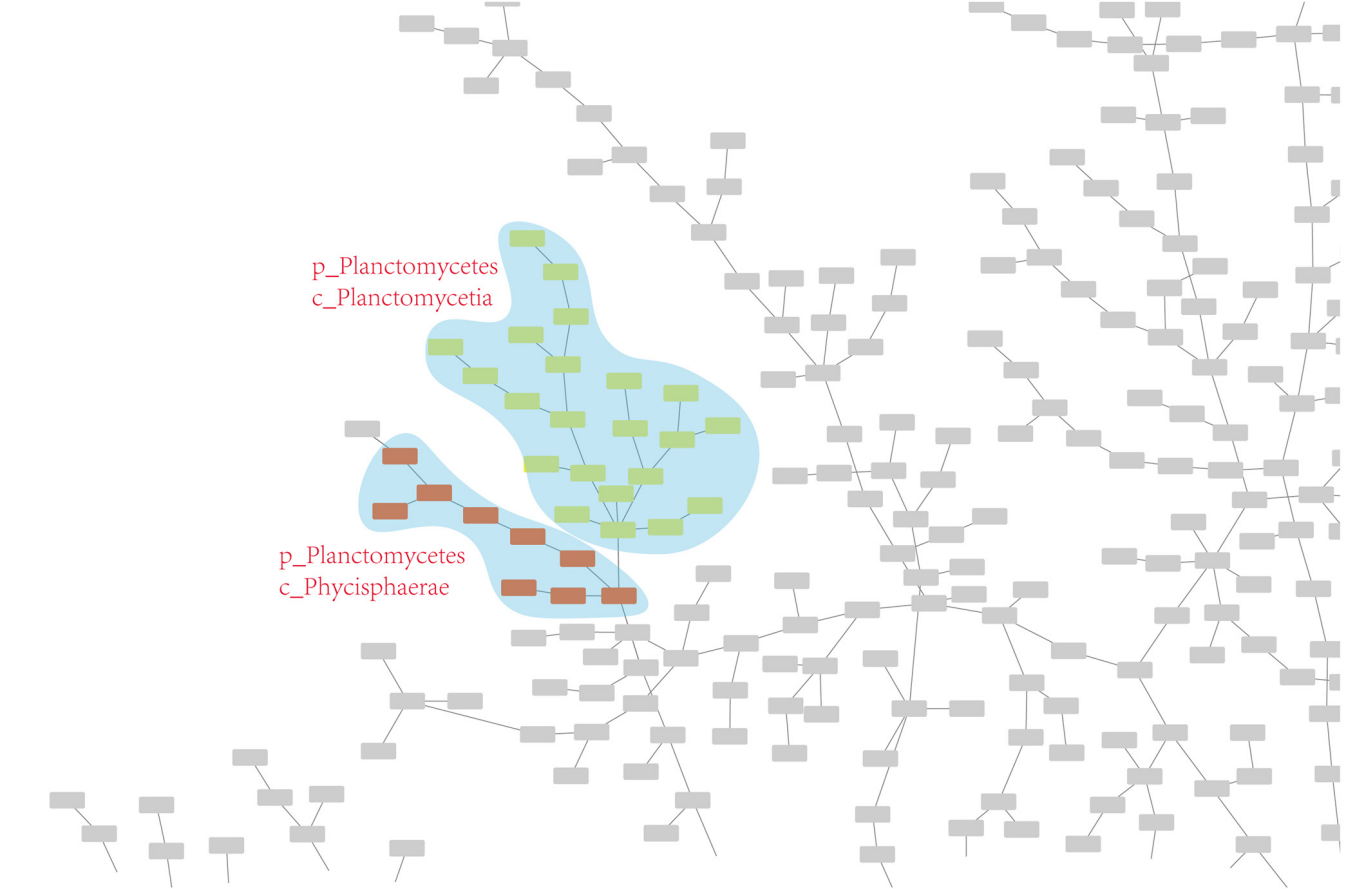
MST result that was updated by adding another eight species in the class *Phycisphaerae*.

To decrease the influence of a single example, we randomly included another 8 species (listed in Fig. S3) from *Phycisphaerae* and show the result obtained with the org_ja method in Fig. 5. In this figure, *Phycisphaerae* and *Planctomycetia* are clustered together, which matches the NCBI taxonomy very well.

In the past, *Planctomycetes* has contained only one class, namely, *Planctomycetia* (18). Some species were promoted to form a new class, *Phycisphaerae*, because they reproduce by binary fission, which is different from the other species’ budding reproduction (20, 21).

### Proteobacteria.

One thousand one hundred forty examples of the *Proteobacteria* are included, and only 1 species (*Glaciecola amylolytica*) is distantly isolated from the main phylum in the MST, connecting to *Bacteroidetes* (*Flavobacteriia*). As for the other 1,139 examples, they are either connected with each other or located very near each other in the MST. Since no other *Glaciecola amylolytica* example is uploaded in the NCBI database to support further research, this situation remains a problem to be solved.

### Conclusion.

In this paper, we propose a standardized 3-step framework based on the protein domain, which includes various candidate models to fulfill difference classification requirements. By applying it to species from the bacterial kingdom, we came to the conclusion that the sequence of domains in a protein and the presence (instead of the presence frequency) of domains play more important roles in determining phenotypes and matching current taxonomy. Finally, we discuss the mismatch of classification results with current taxonomy and list supporting observations from many related works which also validate our proposed framework.

### Future work.

Our proposed framework can be fully automatized, which will motivate us to establish a website or software in future work, facilitating research on species from the domain perspective. In addition, in this paper, we offered an example only in the area of the bacterial kingdom. Actually, the framework can be applied to various contexts, like the family level or the genus level, to carry out classification and investigate species’ phenotypic and genotypic differences in terms of protein domains.

## MATERIALS AND METHODS

**FIG 6:**
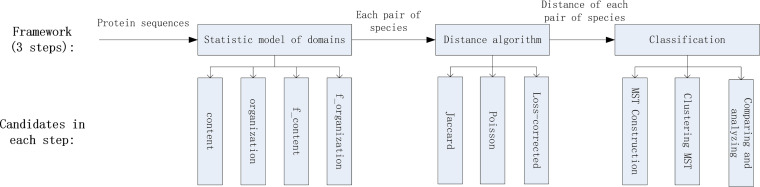
The 3-step process in our proposed framework and candidates in each step.

As shown in Fig. 6, our proposed framework consists of three steps, which involve statistical models of domains, distance models, and the classification process. The statistical models of domains collect the domain information for species according to the pfam data set (22). The distance models are utilized to define the distance of each pair of species according to the statistical results of domains. Finally, the MST is constructed based on the distance results, according to which the classification and analysis will be conducted. Codes relevant to these three steps can be found on GitHub (https://github.com/wr-sky/Domain-Bac-Tax; file names: 4-tree, 5-taxonomy, 6-clustering).

### Statistical models of domains.

**FIG 7:**
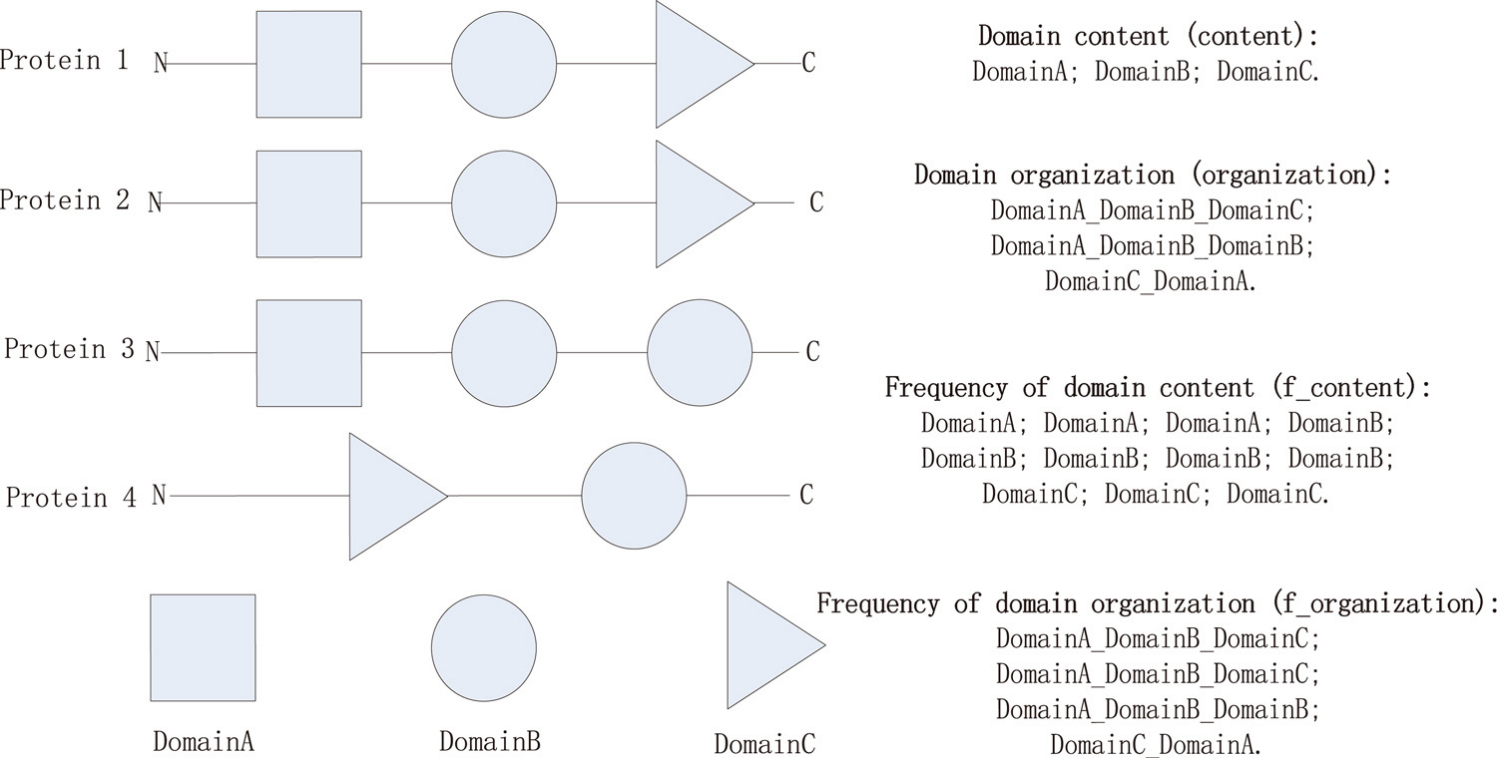
Example of domains in one species and the corresponding records obtained separately by four different statistical models.

In the first step, we propose four statistical models of domains. As shown in Fig. 7, the “content” model records the domain content in the species, which emphasizes the importance of the individual function of each domain and considers only the presence or absence of a domain. The “organization” model takes the sequence of domains in a protein into consideration. Unlike the “content” model, it focuses on a whole protein sequence and, thus, the cofunction of all domains in the protein.

In addition, by considering the presence frequency of the “content” or “organization” in the species, we additionally involve two other statistical models, namely, the “f_content” model and the “f_organization” model, corresponding to the “content” model and the “organization” model. (i) By comparing the “content” and “organization” models, we aimed to determine whether the single domain or the domain organization influences the classification, taxonomy, and phenotypes to the greatest extent. (ii) The utilization of frequency aims to explore the influence of the number of occurrences of a particular domain or domain organization. That is, it attempts to answer the question of which is more important for determining classification and phenotypes, the presence of the content or organization, or the frequency of the content or organization.

To reduce the influence of redundant information on the final classification result, other models such as the one that considers both domain content and organization are not considered model candidates.

### Distance models.

In the second step, we utilize the distance models that calculate the distance of each pair of species based on the statistical results above. Three methods which are commonly utilized in this area and can reflect the biological significance from different perspectives (7, 9) are compared and discussed.

**FIG 8:**
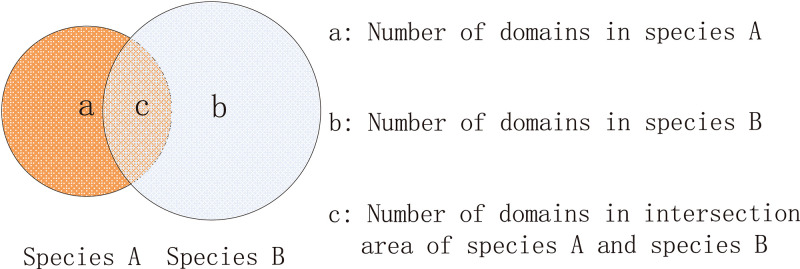
Venn diagram showing the concept of the corresponding three parts when comparing the domains of two species.

The first distance model, Jaccard distance, is shown in equation 3. Parameters *a*, *b*, and *c* represent the numbers of domains in species A, in species B, and commonly in species A and B, respectively, as shown in Fig. 8. The concept of domain here could represent domain content or domain organization.

(3) $$\text{Jaccard distance}=1\;-\;\frac{c}{a\;+\;b\;-\;c}=\frac{a\;+\;b\;-\;2c}{a\;+\;b\;-\;c}$$

Jaccard distance is a very common method to calculate the similarity/differences of two sets. In our scenario, this method deduces the distance of two species under the assumption that the change to a domain (by mutation, loss, or recombination) happens randomly and independently.

The second distance model, Poisson distance, is shown in equation 4. Unlike Jaccard distance, it works under the assumption that the change of domain follows the Poisson process (9). $-\text{ln}\frac{c}{a}$ and $-\text{ln}\frac{c}{b}$ represent the distances between the two species and their ancestor. The distance between the two species is further defined as the geometric mean of the distances to their common ancestor.

(4) $$\text{Poisson distance}={\left(\text{ln}\frac{c}{a}\times \text{ln}\frac{c}{b}\right)}^{1/2}$$

The third distance model, loss-corrected distance, is shown in equation 5. It considers the possibility of massive gene loss during evolutionary history. Thus, to reduce its influence, the distance is corrected by utilizing the smaller domain set as the comparison standard. The distances calculated by the three models range from 0 to 1, with 1 implying the greatest distance and 0 the smallest.

(5) $$\text{loss-corrected}\;\text{distance}=\{\begin{array}{c} \frac{a\;-\;c}{a},a\;\leq \;b\\ \frac{b\;-\;c}{b},a\;>\;b \end{array}$$

Four statistical models together with 3 distance models yield 12 different combination candidates, which offer flexibility for different scenarios.

### Classification.

**FIG 9:**
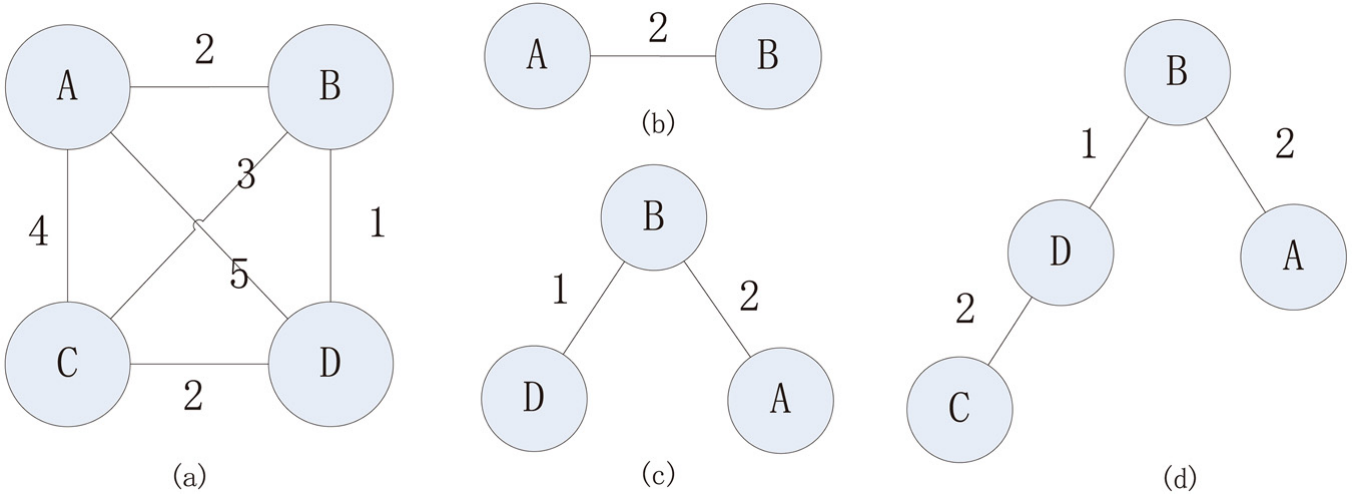
Example of how to construct the MST based on the fully connected network. (a) A fully connected network with distances marked. (b) A is first selected and then its nearest node, B. (c) D has the nearest distance to A or B compared with C; thus, D is involved in the MST. (d) C is nearest to D; thus, C is connected to D.

In the third step, the MST is constructed by the distance results described above by Prim’s algorithm (Fig. 9). In the algorithm, first, one species is randomly selected as the initial node of the MST. Then, another node is involved in the MST, which has minimal distance from a node that is already in the MST, and these two nodes are connected. The second step is repeated until all nodes are included in the MST. According to the Prim’s algorithm, the MST algorithm will find a pair of nodes with minimal distance in each step, which, in our scenario, represented the smallest differences between domains.

**FIG 10:**
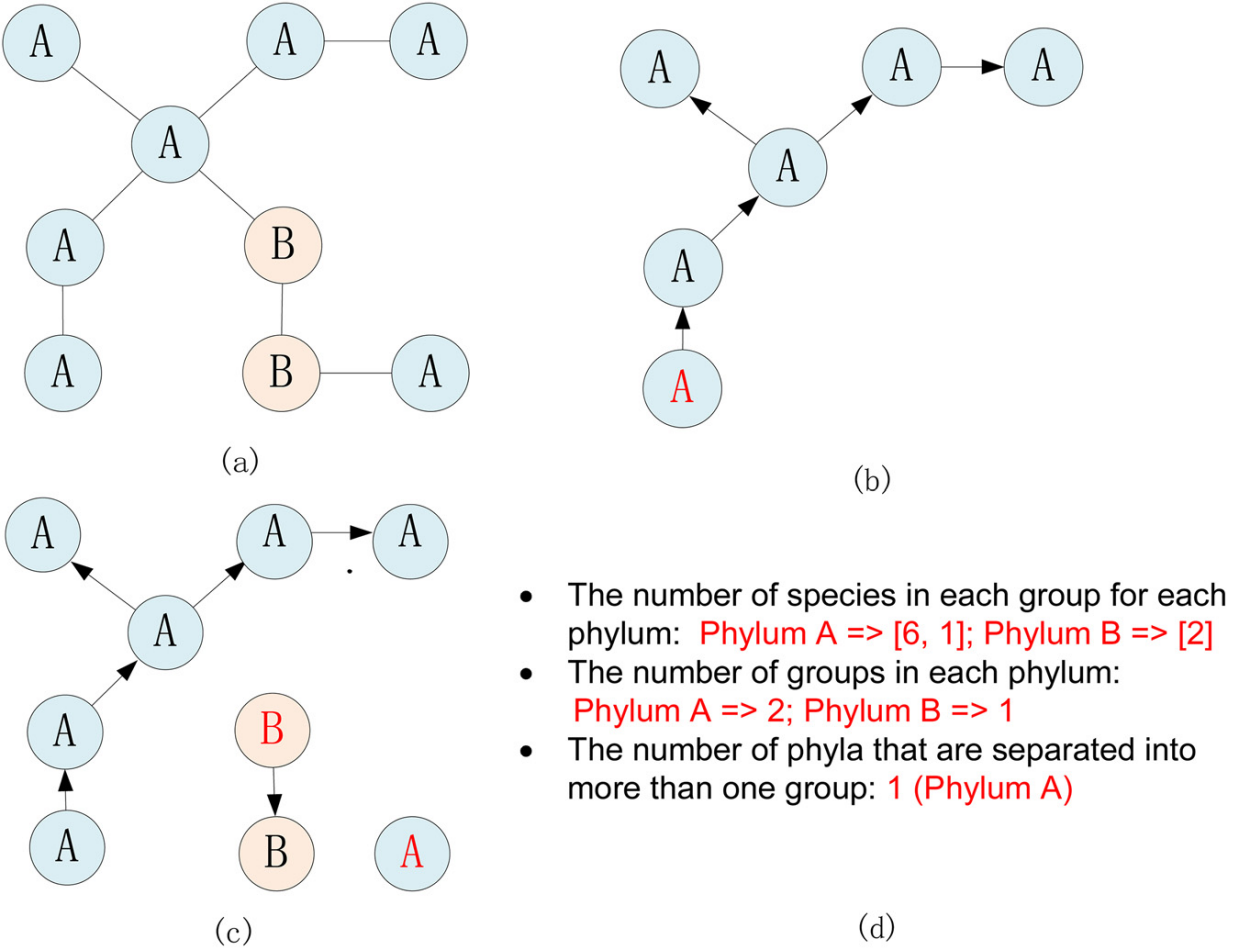
An example of clustering the MST results. (a) Nodes are tagged according to the taxonomy at the phylum level (phyla A and B in this example) (b) One node (in red) is randomly selected, and the connected nodes are iteratively searched with the same phylum and involved in the group. (c) The second step is repeated until all nodes are grouped. (d) Statistical results are calculated.

Then, as shown in Fig. 10, we wrote a program to cluster and compare the MST result according to the current taxonomy standards to further analyze phenotypic indifferences, which mainly focuses on three aspects: (i) the number of species in each group for each phylum, (ii) the number of groups in each phylum, and (iii) the number of phyla that are separated into more than one group.

In the algorithm, first, all nodes are tagged according to the taxonomy standard at the phylum level (phyla A and B in this example). Second, one node is randomly selected, and the neighboring nodes are iteratively searched with the same phylum and involved in the group. Third, the second step is repeated until all nodes are grouped. Finally, the statistical results are calculated as illustrated above.

### Implementation.

By applying the framework to the bacterial kingdom, we show one of its implementation examples. Some valuable conclusions can be summarized, and the validation of our proposed framework can also be verified. Codes relevant to the implementation can be found on GitHub (https://github.com/wr-sky/Domain-Bac-Tax; file names: 1-download, 2-checkm, 3-pfam).

The genome sequences of the bacterial domain were downloaded according to the metadata (7 February 2021) on NCBI (ftp://ftp.ncbi.nlm.nih.gov/genomes/refseq/bacteria/assembly_summary.txt). Initially, 205,791 species were recorded in the text file. For a more credible result, we selected the sequences with the entries “complete genome” in the “assembly_level” column, “latest” in the “version_status” column, “full” in the “genome_rep” column, and “representative genome” or “reference genome” in the “refseq_category” column, filtering out 2,587 sequences for analysis. Then, we downloaded these 2,587 sequences in faa (FASTA amino acid) and fna (FASTA nucleic acid) formats from NCBI. The quality of each sequence was inspected by the CheckM program with the standard shown in equation 6.

(6) quality=completeness−(5×contamination)

Nineteen sequences with quality results under 95% were removed, and thus, 2,568 genome sequences were utilized for further analysis. Their information can be found in Data Set S1. In addition, 6 species from the domain *Archaea* were randomly selected and involved as the external species: Desulfurococcus amylolyticus, Halorhabdus utahensis, Halomicrobium mukohataei, Halogeometricum borinquense, Nitrososphaera viennensis, and Saccharolobus solfataricus.

The 2,568 nucleic acid sequences in FASTA format were analyzed by the pfam_scan.pl program with default settings (22). The results, in a csv-format output file, listed the possible domains in each sequence. Domains with overlapping regions were polished by selecting the domain with the maximal bit score (https://github.com/wr-sky/Domain-Bac-Tax/tree/main/7-data/pfam_tophit). Then, the 2,568 bacterial protein domain results were processed by our proposed framework above.
