# Supplementary material for: A Standardized Framework for Better Understanding of Phenotypic Differences within Bacterial Phyla Based on Protein Domain
Source: J Bacteriol. 2022 Jun 2;204(6):e00141-22. doi: 10.1128/jb.00141-22 (PMC9210965; doi:10.1128/jb.00141-22)
Supplement: Supplemental File 1 — Fig. S1-S12; Tables S1-S2. Download jb.00141-22-s0001.pdf, PDF file, 1.4 MB [file jb.00141-22-s0001.pdf]

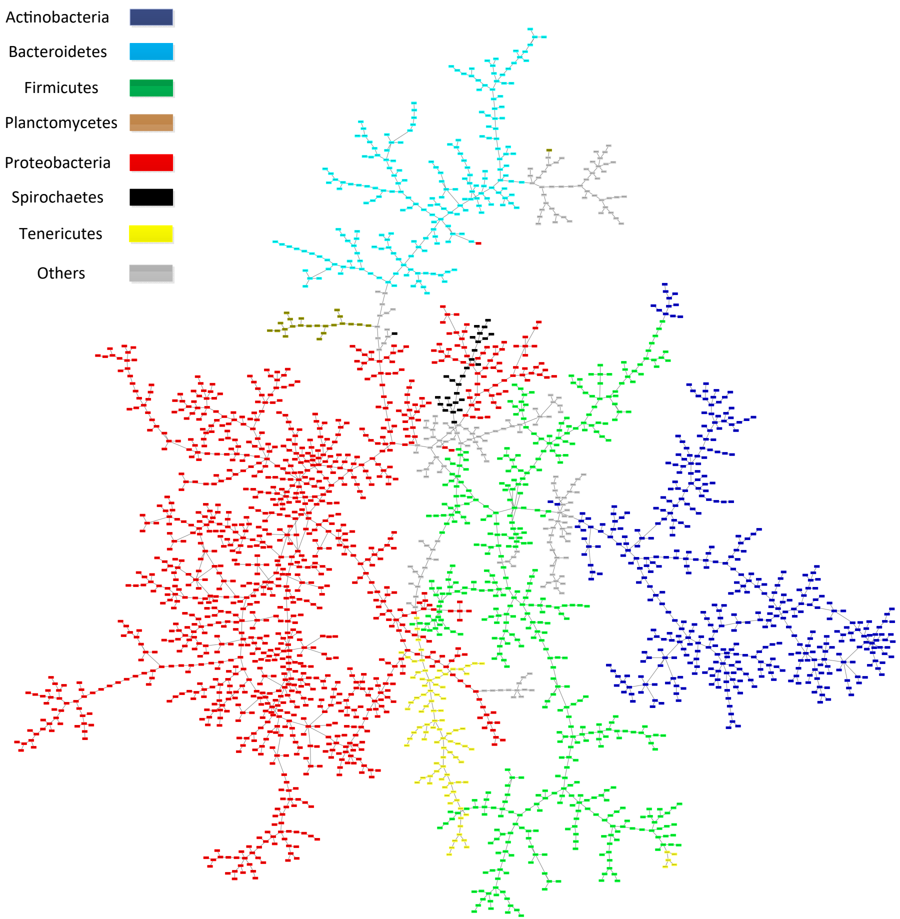

Supplementary Information Figure S1. MST result by "content" model and Jaccard distance.

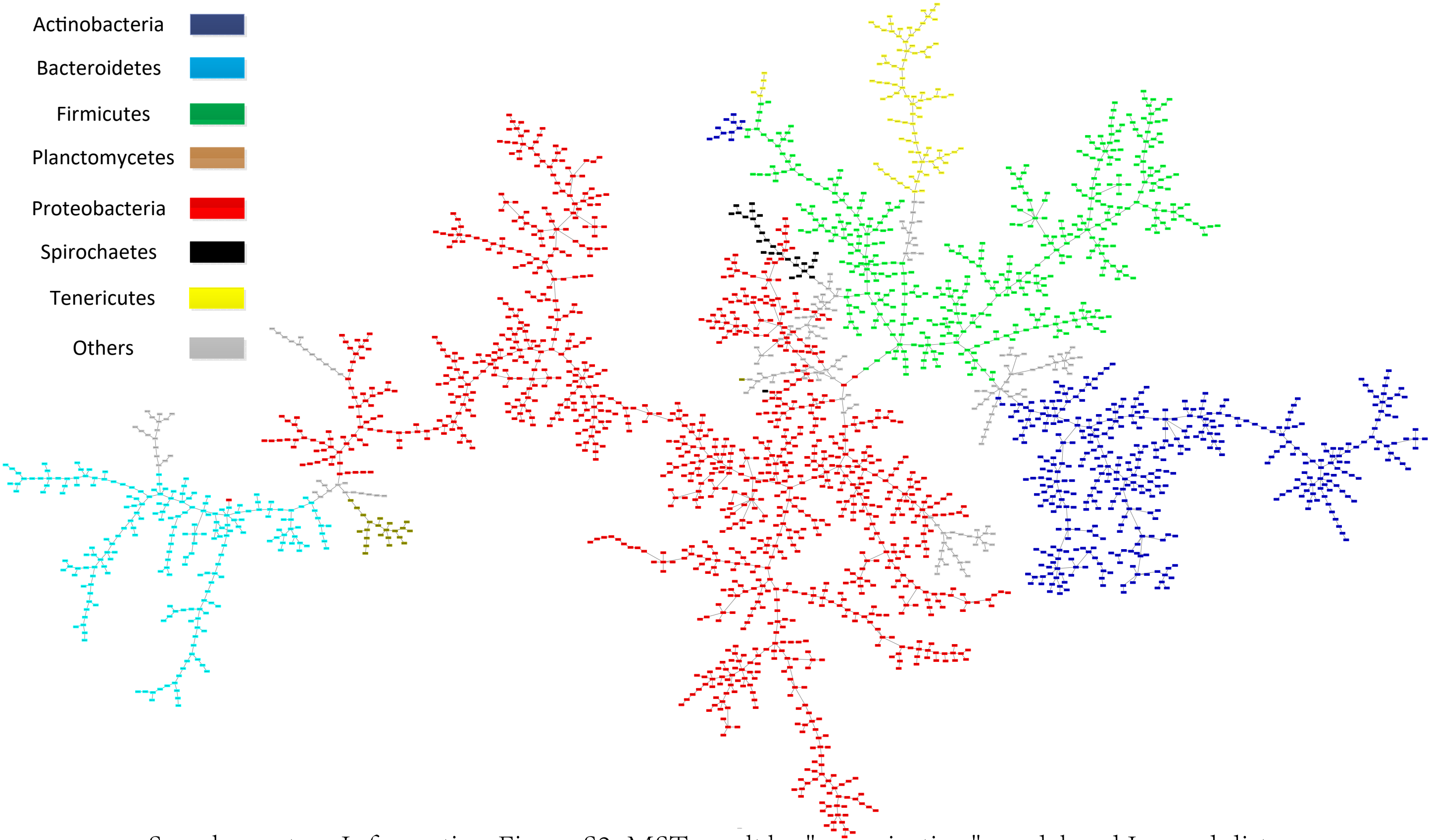

Supplementary Information Figure S2. MST result by "organization" model and Jaccard distance.

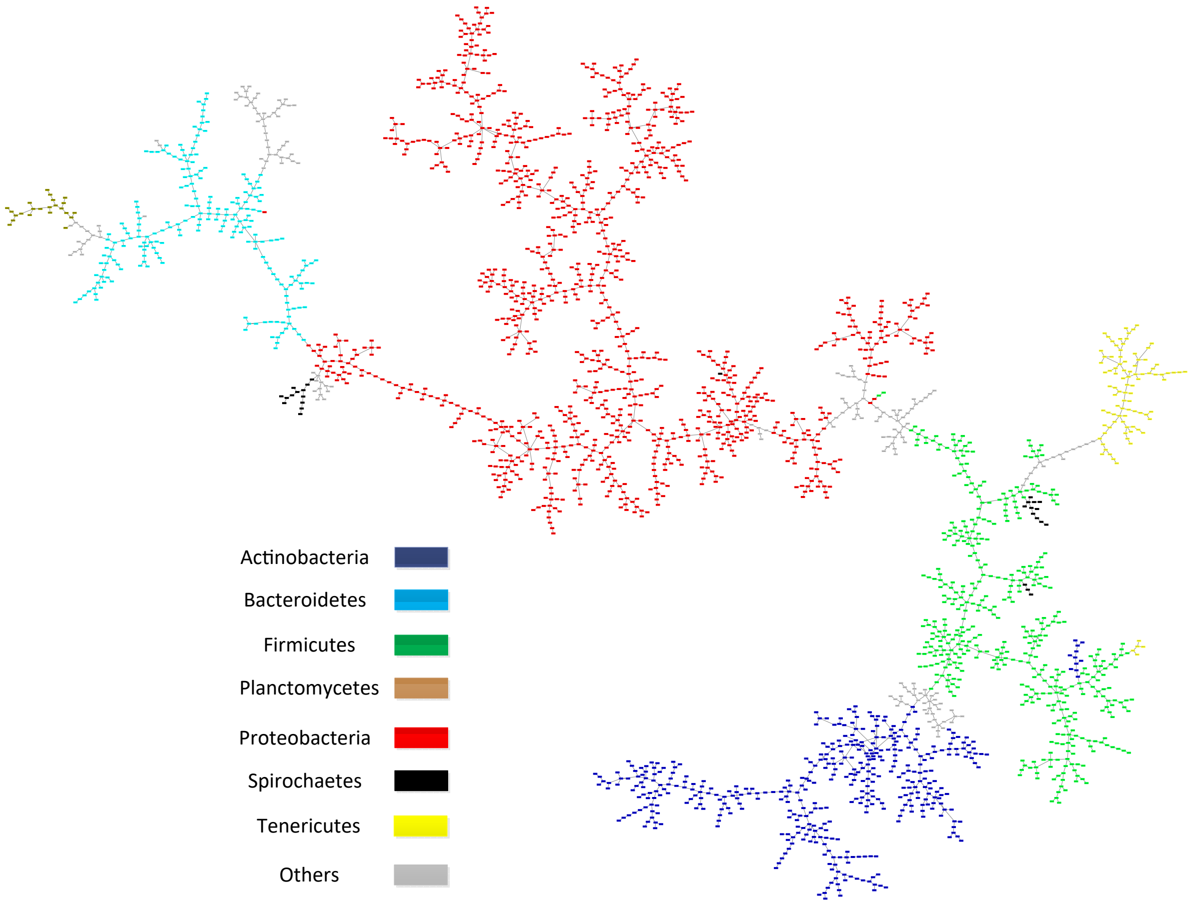

Supplementary Information Figure S3. MST result by "f_content" model and Jaccard distance.

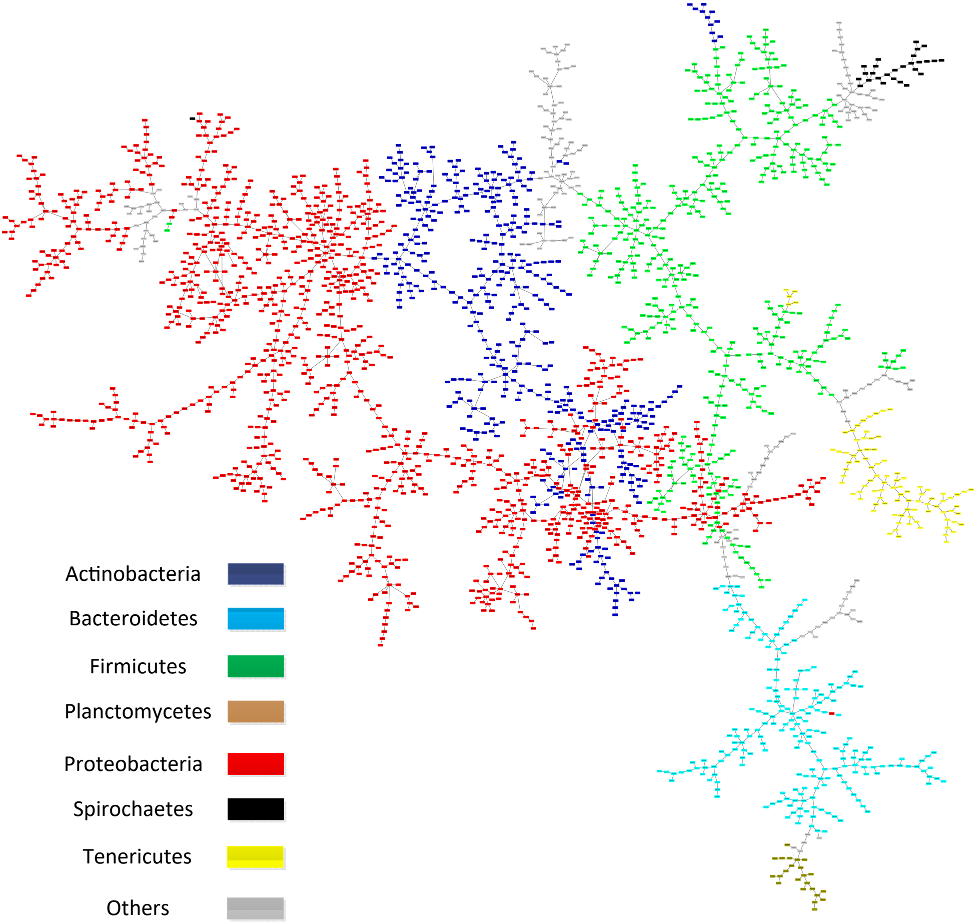

Supplementary Information Figure S4. MST result by "f_organization" model and Jaccard distance.

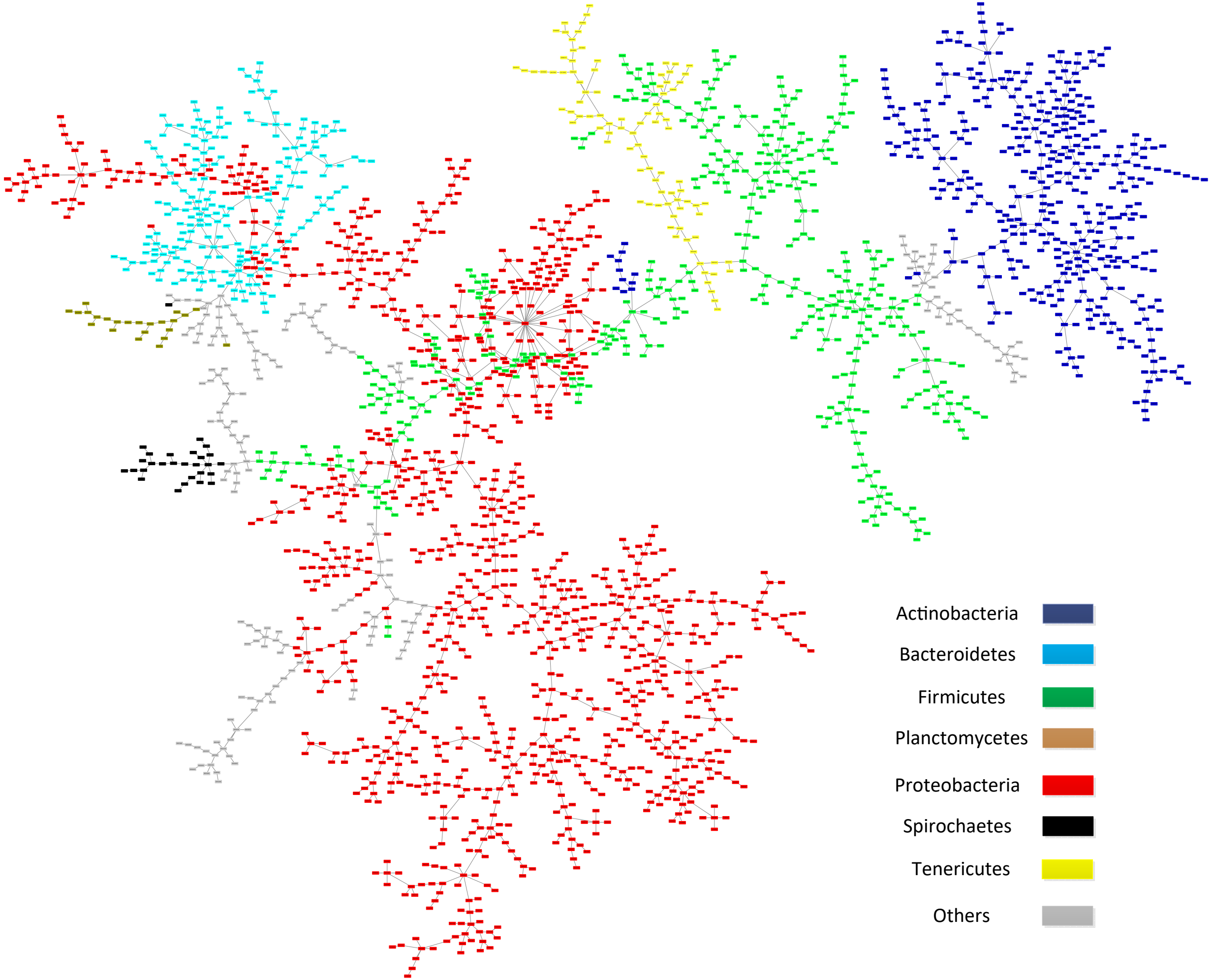

Supplementary Information Figure S5. MST result by "content" model and Poisson distance.

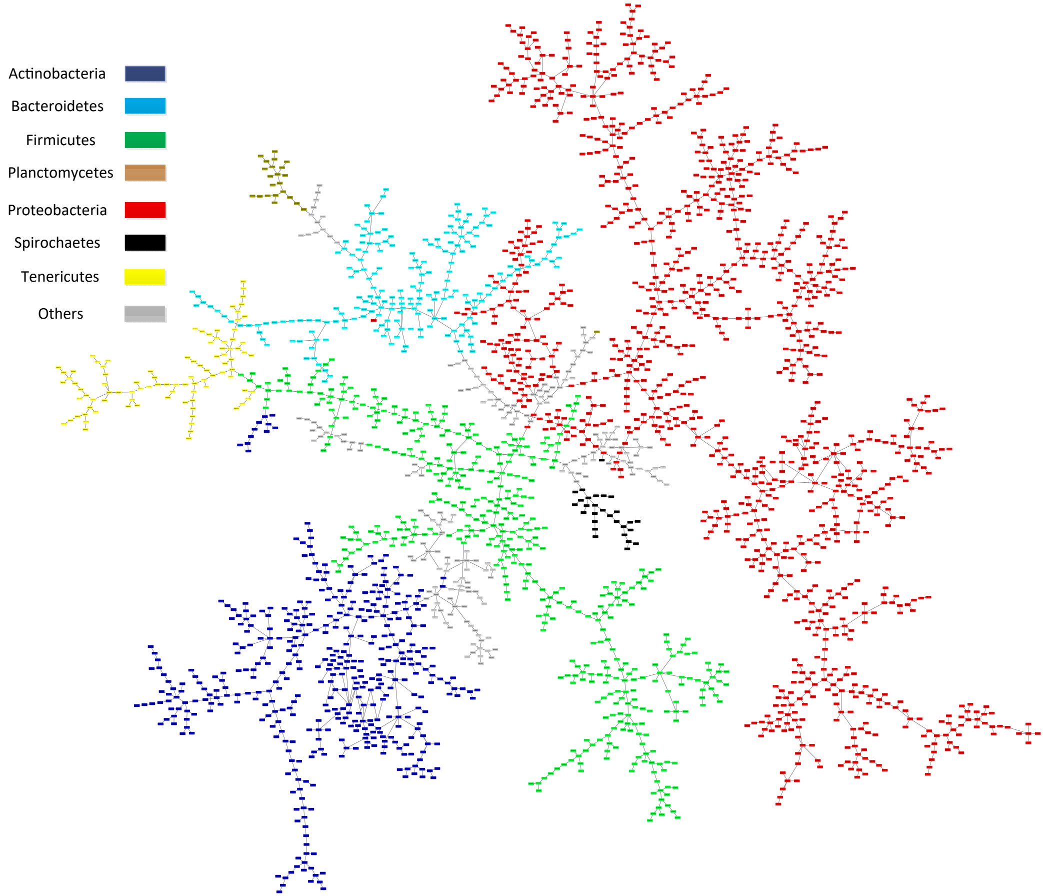

Supplementary Information Figure S6. MST result by "organization" model and Poisson distance.

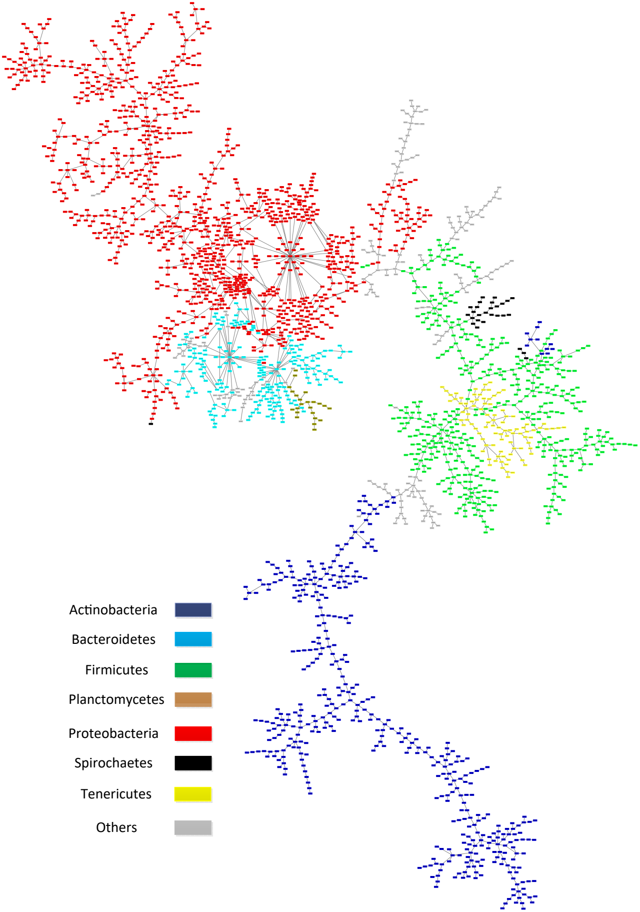

Supplementary Information Figure S7. MST result by "f_content" model and Poisson distance.

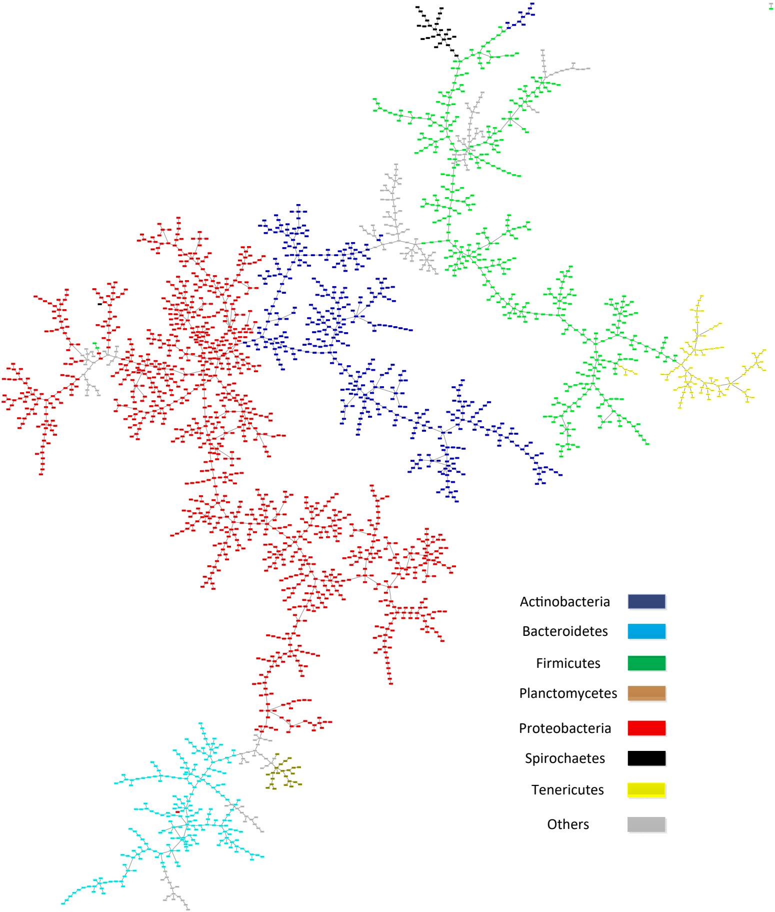

Supplementary Information Figure S8. MST result by "f_organization" model and Poisson distance.

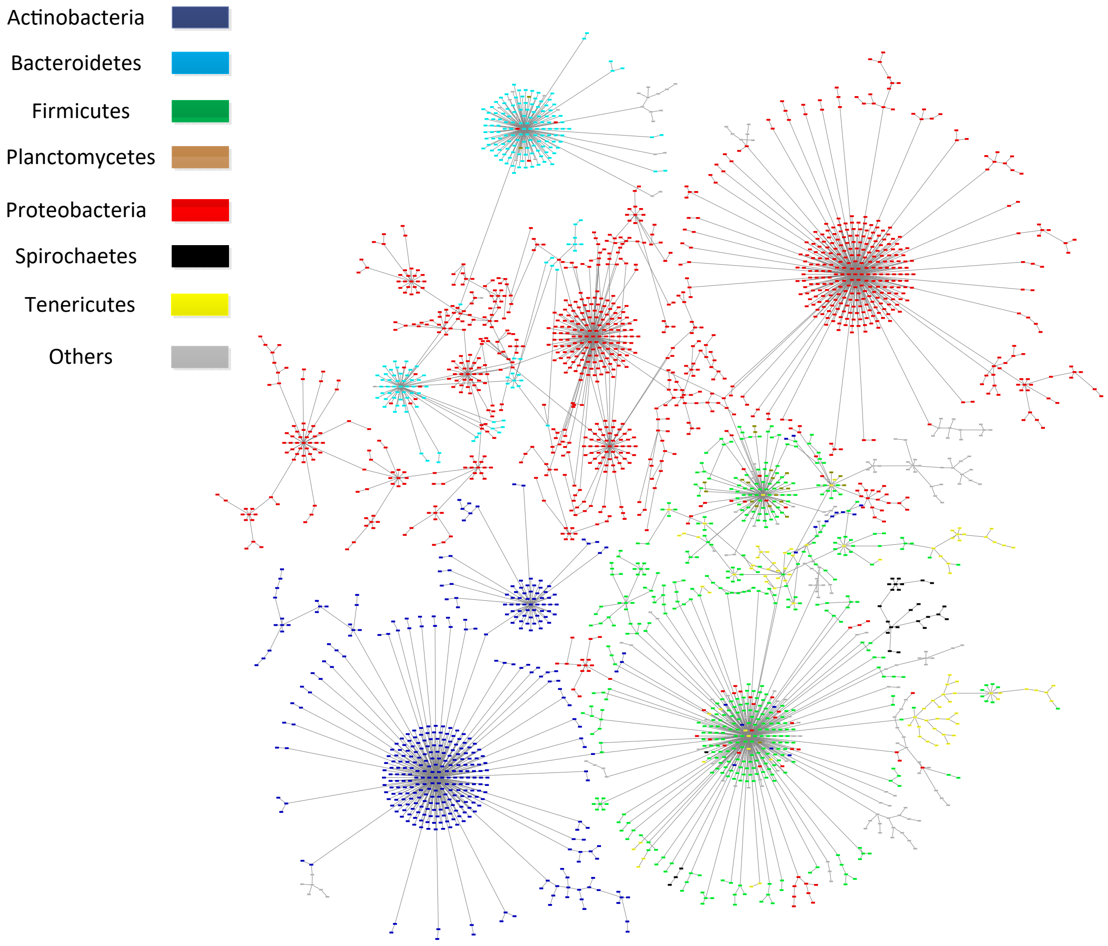

Supplementary Information Figure S9. MST result by "content" model and Loss-corrected distance.

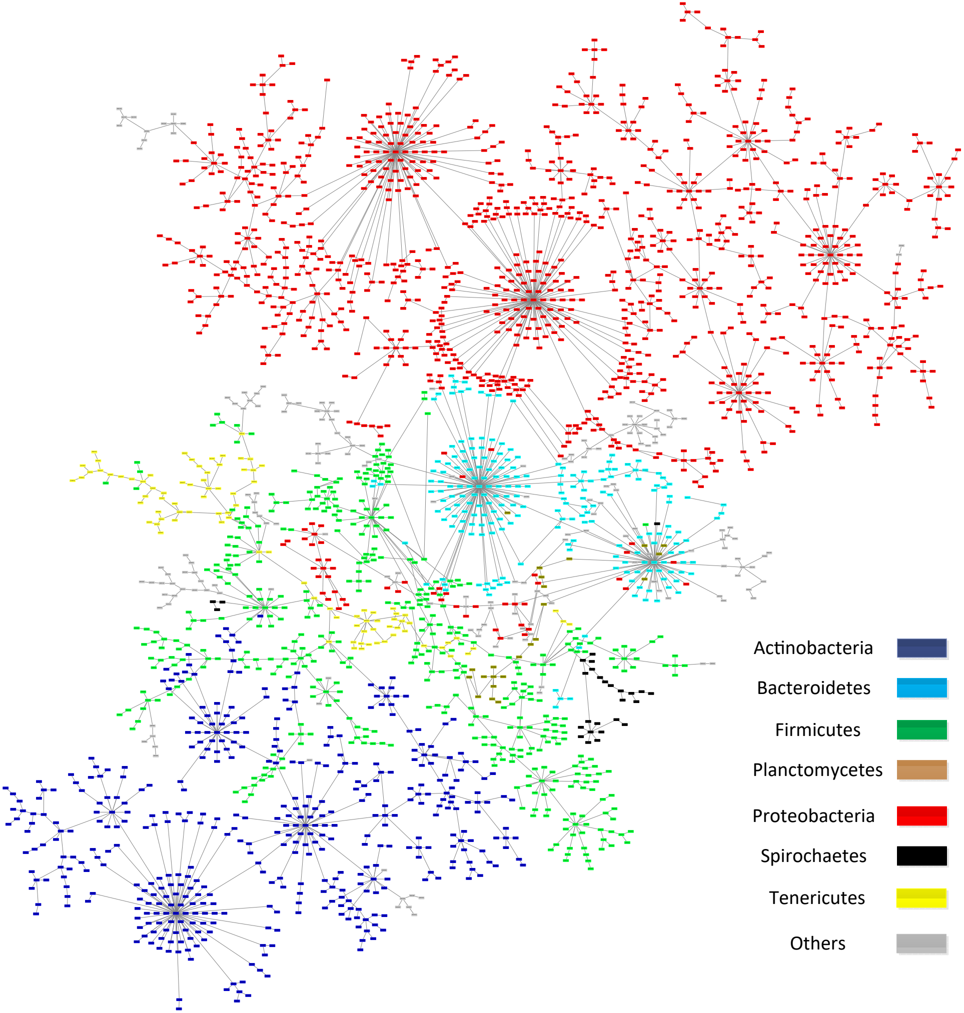

Supplementary Information Figure S10. MST result by "organization" model and Loss-corrected distance.

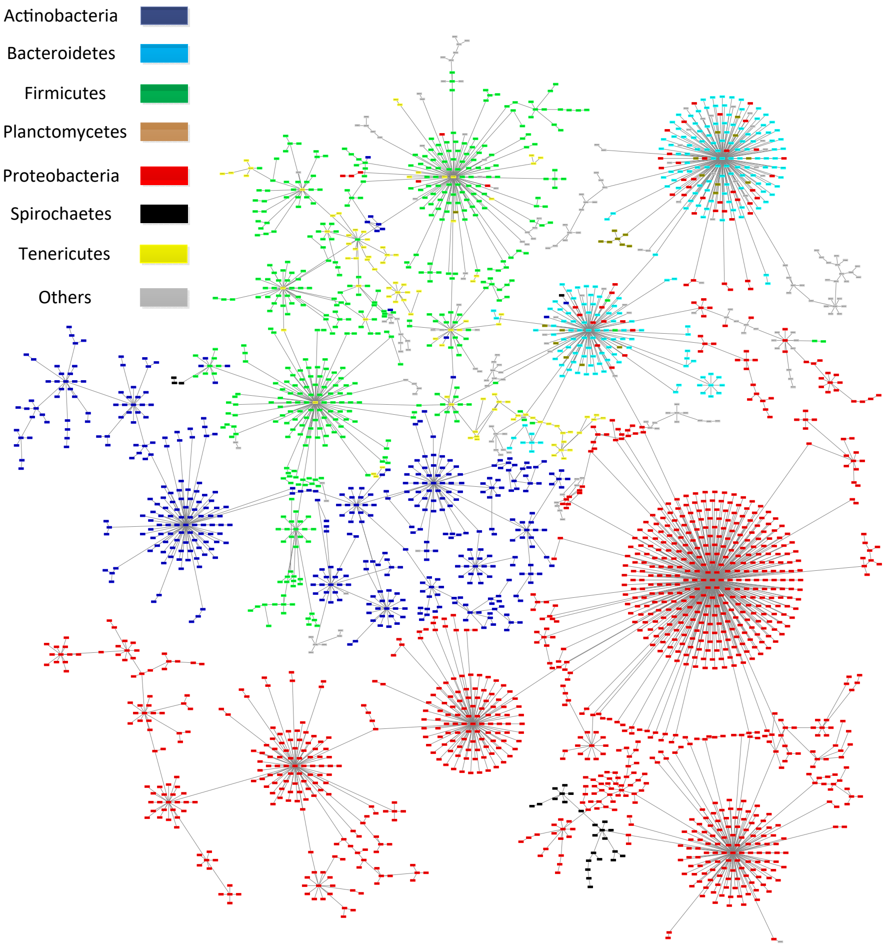

Supplementary Information Figure S11. MST result by "f_content" model and Loss-corrected distance.

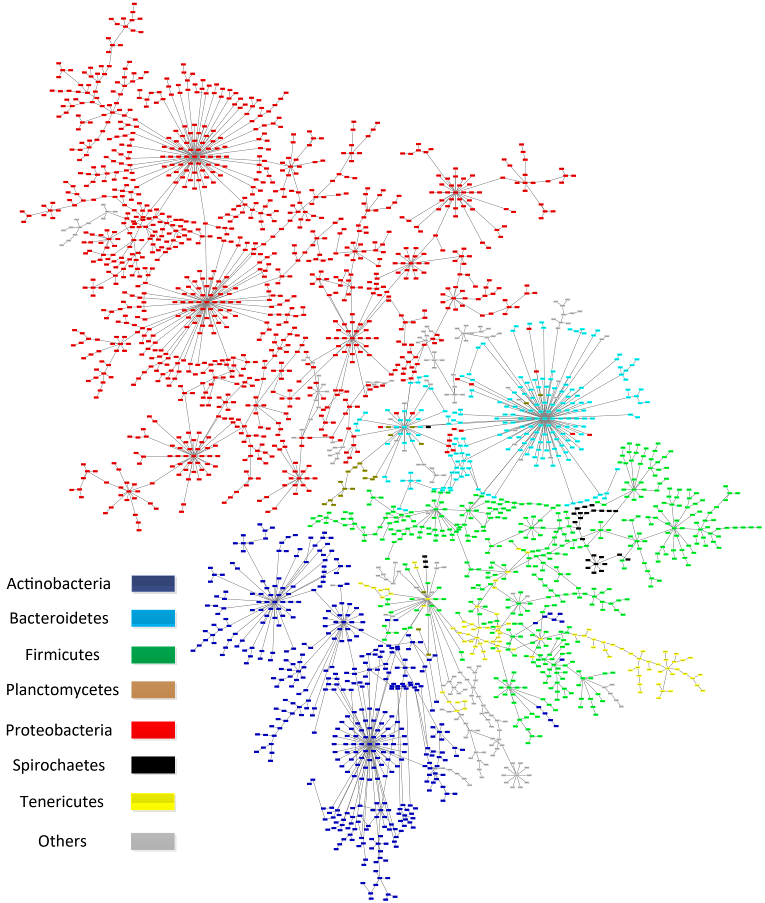

Supplementary Information Figure S12. MST result by "f_organization" model and Loss-corrected distance.

Supplementary Information Table S1: Phyla classification results by Jaccard and Poisson distance models.

	Phylum	total	con_ja	org_ja	f_con_ja	f_org_ja	con_po	org_po	f_con_po	f_org_po	common
1	Acidobacteria	9	1		1		1		1	1	
2	Actinobacteria	432	2, 11	2, 11	11	2, 11	2, 11	2, 11	11	2, 11	11
3	Aquificae	8									
4	Archaea	6									
5	Armatimonadetes	1									
6	Bacteroidetes	212				1					
7	Caldiserica	1									
8	Calditrichaeota	1									
9	Chlamydiae	12									
10	Chlorobi	11									
11	Chloroflexi	13	3		3	3	3	3	1, 3	3	3
12	Coprothermobacterota	1									
13	Cyanobacteria	28									
14	Deferribacteres	5									
15	Deinococcus_Thermus	24									
16	Dictyoglomi	2									
17	Elusimicrobia	2									
18	Firmicutes	458	2		2, 3, 9	2, 9	2, 4, 150		2, 4, 142, 150	2	2
19	Fusobacteria	15									
20	Gemmatimonadetes	3									
21	Ignavibacteriae	2									
22	Kiritimatiellaeota	1									
23	Nitrospirae	1									
24	Planctomycetes	23	1	1	1	1	1	1	1	1	1
25	Proteobacteria	1140	1, 2, 20, 74	1, 2, 12, 20, 27, 74	1, 2, 5, 53, 75	1, 2, 12, 20, 27, 74	1, 1, 1, 2, 3, 23,27	1, 2, 5, 12, 15, 27, 74	1, 2, 3, 23, 29	1, 2, 5, 27, 27, 74	1, 2
26	Spirochaetes	28	1	1	1, 3, 11	1	1	1	1, 3	1	1
27	Synergistetes	5									
28	Tenericutes	94	5	5	5	5		5		5	5
29	Thermodesulfobacteria	6									
30	Thermotogae	23									
31	Verrucomicrobia	7	2		2, 2	3	2		1, 2, 2	2	2

Supplementary Information Table S2: Phyla classification results by Loss-corrected distance model.

	Phylum	con_le	org_le	f_con_le	f_org_le
1	Acidobacteria	7	4	8	4
2	Actinobacteria	13	5	11	5
3	Aquificae	4	2	4	3
4	Archaea	1	1	1	1
5	Armatimonadetes	1	1	1	1
6	Bacteroidetes	1	2	1	1
7	Caldiserica	1	1	1	1
8	Calditrichaeota	1	1	1	1
9	Chlamydiae	1	1	1	1
10	Chlorobi	2	2	3	2
11	Chloroflexi	10	7	9	6
12	Coprothermobacterota	1	1	1	1
13	Cyanobacteria	1	1	3	1
14	Deferribacteres	5	1	5	2
15	Deinococcus_Thermus	4	1	4	1
16	Dictyoglomi	1	1	1	1
17	Elusimicrobia	2	2	2	2
18	Firmicutes	307	53	291	37
19	Fusobacteria	9	2	6	2
20	Gemmatimonadetes	3	2	3	1
21	Ignavibacteriae	2	2	2	2
22	Kiritimatiellaeota	1	1	1	1
23	Nitrospirae	1	1	1	1
24	Planctomycetes	21	10	17	7
25	Proteobacteria	51	18	57	22
26	Spirochaetes	3	3	3	3
27	Synergistetes	4	1	4	1
28	Tenericutes	23	2	22	4
29	Thermodesulfobacteria	1	1	1	1
30	Thermotogae	5	1	5	3
31	Verrucomicrobia	5	5	6	5
